# Comparison of freehand technique and a novel laser-guiding navigation system in femoral neck-cannulated screw fixation: a randomized controlled trial

**DOI:** 10.1186/s12893-023-02226-w

**Published:** 2023-10-23

**Authors:** Hua Gao, Zhenyu Liu, Xiaodong Bai, Gang Wang, Guoqiang Xu, Ji Ma, Yijun Wang, Jiatian Wang, Wentao Chen, Baojun Wang

**Affiliations:** Department of Orthopaedics, Beijing Friendship Hospital, Capital Medical University, Beijing, China

**Keywords:** Femoral neck fracture, Cannulated screw fixation, Laser-guiding navigation, Freehand technique

## Abstract

Cannulated screw fixation is essential in treating femoral neck fractures, and the widely used freehand technique has several limitations. Therefore, we designed a new laser-positioning and navigation system and compared its efficacy with that of the traditional freehand technique in the cannulated screw fixation of femoral neck fractures. This randomized controlled single-blind trial recruited patients with femoral neck fracture, who were treated using either the newly designed laser-navigation device or the freehand technique. In in-vitro experiments, using the femoral neck model, the laser group was better than the freehand group in terms of operation time (*P* = 0.0153) and radiation exposure time (*P* < 0.001). In in-vivo experiments, involving 30 patients (15 in each group), the laser group was better than the freehand group in terms of operation time (*P* < 0.001), radiation exposure time (*P* < 0.001), blood loss (*P* < 0.001) and first success rate (*P* = 0.03). There was no difference in visual analog scale score, Harris score, and fracture-healing time between the two groups. In conclusion, the novel laser-guiding navigation system resulted in shorter operation time, less radiation exposure, and higher first success rate compared with the freehand technique. Further qualified investigations with a larger number of patients and longer follow-up are required in the future.

## Introduction

 Femoral neck fractures are very common and often result in obvious morbidity and mortality. Almost 2.4 million femoral neck fractures occur annually worldwide [1]. According to the three-point principle, the inverted triangular cannulated screw is the classical method for treating femoral neck fractures [2].

Numerous novel technologies, such as orthopedic surgical robot navigation, computer-aided navigation, and orthopedic minimally invasive intelligent visualization system, have been invented to optimize the process of channel screw placement [3–5]. However, including the most widely used freehand method, these technologies cannot satisfy the needs of simplicity, accuracy, and low radiation exposure simultaneously [6].

Based on Desargues’s theorem [7] and the characteristics of X-ray projection [8], and using the “three-line coplanar positioning” method [9], our team integrated the G-arm machine with the self-designed laser navigation device to achieve the effect of visual positioning of the channel-screw path. This system has been proven to be accurate, simple, and to have less radiation exposure through the preliminary in-vitro experiment. This study verifies the safety and effectiveness of using cannulated screw fixation to treat patients with femoral neck fracture, and compares it with freehand methods.

## Materials and methods

**Fig. 1:**
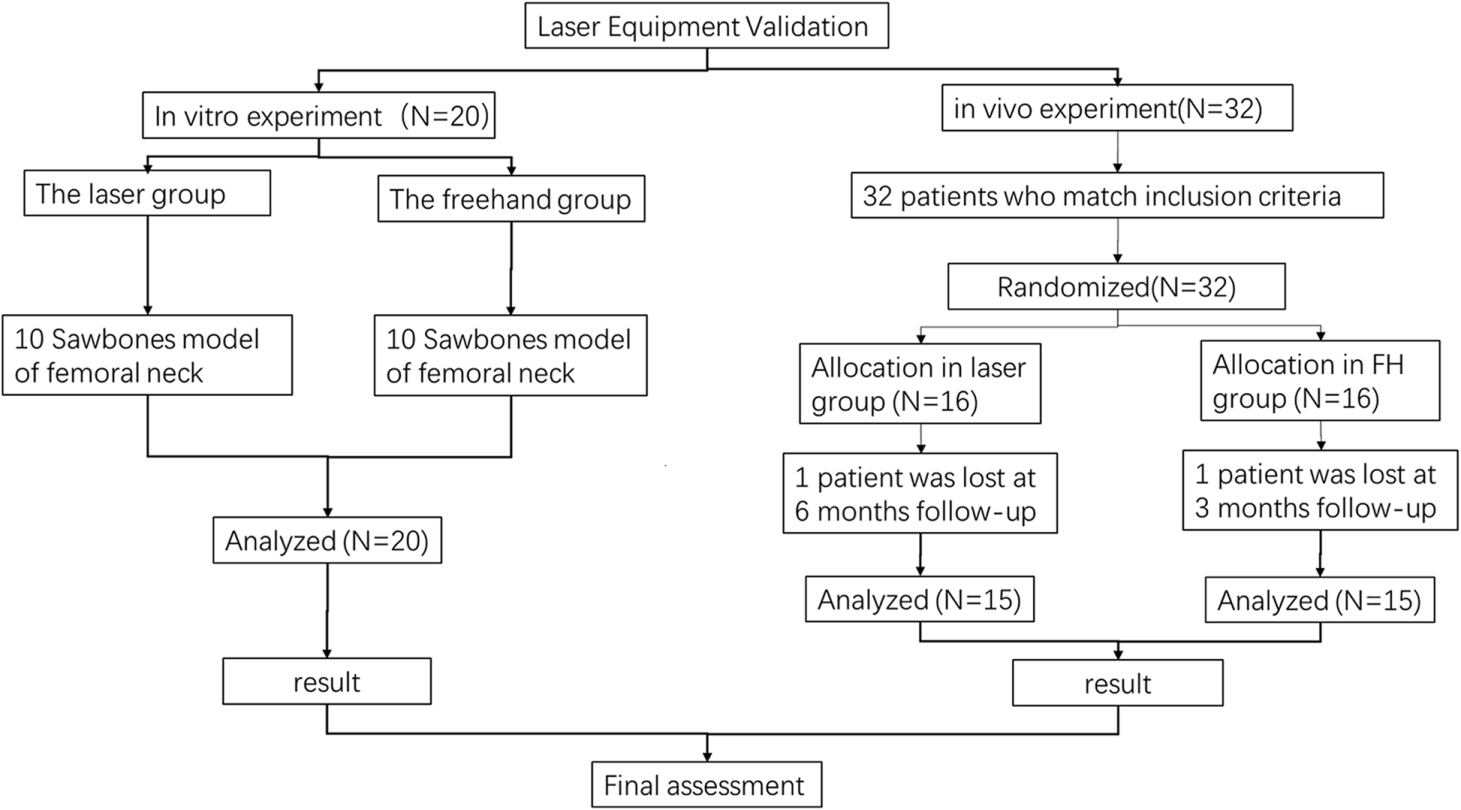
Flowchart of the study design, including in-vivo and in-vitro experiments

In this study, a novel laser navigation device of the femoral neck-channel screw was proposed. Experiments were performed in-vitro and in-vivo to verify its efficacy. Figure 1 shows the flow diagram of the study.

### Description of the novel device

**Fig. 2:**
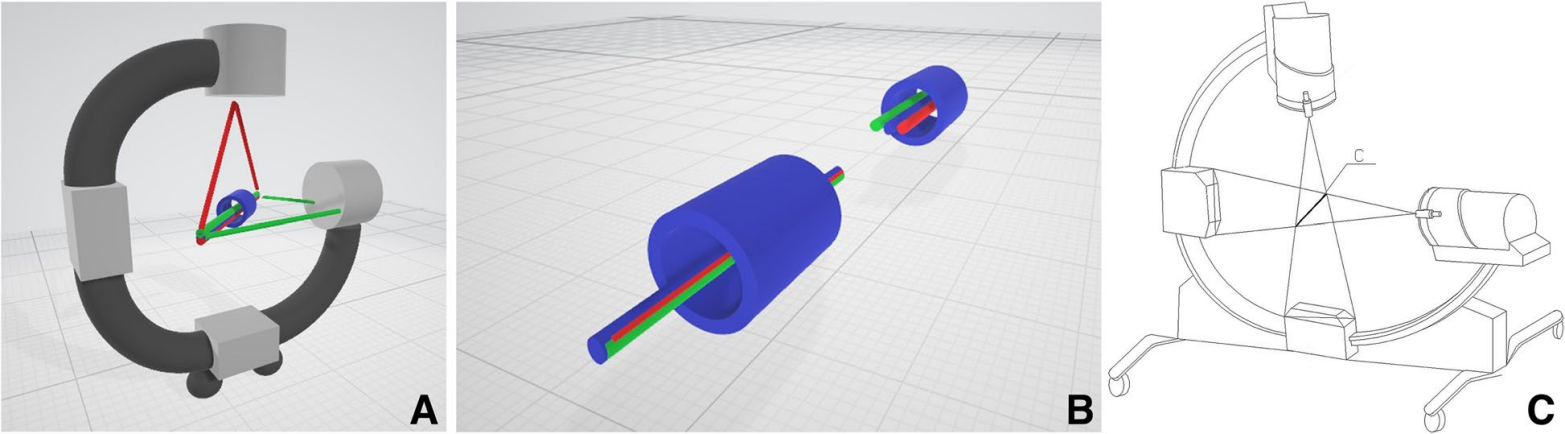
Work principle sketch maps of the laser guiding navigation device (**A**). This meant that the horizontal plane X-ray central fluoroscopy axis of G arm (the green line), vertical plane X-ray central fluoroscopy axis (the red line) and the target screw passage central axis (the blue line) remain coaxial, known as “three-line coaxial” (**B**, **C**). The blue tube represented target channel (**B)**

According to Desargues’ theorem of projection geometry and the characteristics of X-ray fluoroscopic imaging, a three-axis co-linear approach (fluoroscopic center axis, target axis and laser positioning axis) is adopted. At the same time, we make use of the visualization characteristics of the laser, when the laser lines representing two planes (Desargues’ plane) cross each other, the confluence is the target channel, to realize the visualization of the three-dimensional positioning of the target channel. The laser-guiding navigation device is composed of two laser pointers (in-line, energy 100 mW, wavelength 520 nm, wavelength 635 nm, Senwei), special laser pointer holder, and locating ring attached to the G-arm’s image intensifier (G-arm Orca, WHALE MEDCINE, Boston, USA). Before operation, the device was adjusted repeatedly to ensure that the two laser lines, X-ray fluoroscopy center line and the target axis, were completely overlapped in horizontal and vertical planes simultaneously (Fig. 2). When this device was first installed in the G-arm’s image intensifier, it required an adjustment time of approximately 10 min. Each G-arm machine required installation and adjustment for the first time and only required approximately 1 min of calibration for subsequent use, and this should be finished before the operation.

### Laser navigation system in in-vitro experiments

**Fig. 3:**
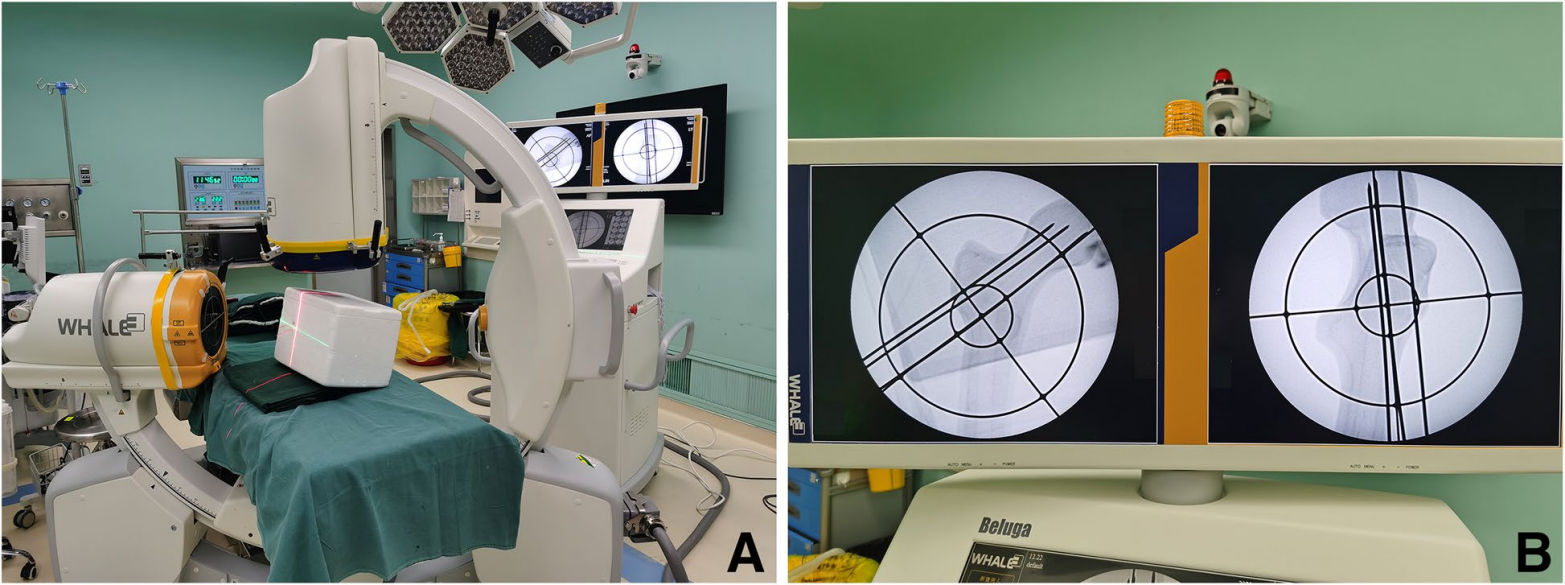
The sawbone femoral neck model was firmly fixed in the blind box (**A**). Ensured that the distal guide pin (Φ = 2 mm) was drilled along the red and green laser lines and confirmed that the Kirschner wire passed through target chanel through fluoroscopy (**B**). The other two guide pins were also placed by the parallel drill guide

In the sawbone femoral neck model in the blind box, the femoral neck cannulated screw-guide pin was placed using a laser-navigation system and freehand methods, respectively (Fig. 3). Both groups adopted the method of three parallel screws. In the freehand group, the distal guide pin was first placed using G-arm fluoroscopy. After the position was confirmed, the other two guide pins were placed using parallel drill guide. In the laser group, after the laser emitter and positioning ring were calibrated, the distal guide pin was inserted first, under the guidance of laser navigation. After the position was confirmed, the other two guide pins were placed using the parallel drill guide. The operations were all performed by the same doctor, and each group contained 10 models. The operation and radiation time were recorded, and the independent sample t test was used for statistical testing.

### Laser navigation system in in-vivo experiments

#### Patients

This study enrolled femoral neck-fracture patients who underwent internal fixation with cannulated screws at Beijing Friendship Hospital (Level I trauma center in the region) from June 1st, 2021 to April 1st, 2022. Inclusion criteria: age > 18 years, close femoral neck fracture, and acute fracture (the time interval from injury to operation < 2 weeks). Exclusion criteria: pathological fracture, open fracture, old fracture, multiple fractures of the ipsilateral femur, secondary surgery, and patients who preferred other operations. Informed consent was obtained from all patients or their legal guardians.

This was a randomized controlled single-blind trial. The patients enrolled were randomized into laser group and freehand group (Fig. 1). Randomization seed was specified, and the randomization sequence was generated using the PROC PLAN procedure of SAS 9.4 (SAS Institute, Cary, NC, USA) software with a 1:1 allocation. All operations were performed by two orthopedists who had received the same amount of time training and had completed more than 50 internal femoral neck fracture fixation by themselves in 2 years.

#### Treatment protocol

**Fig. 4:**
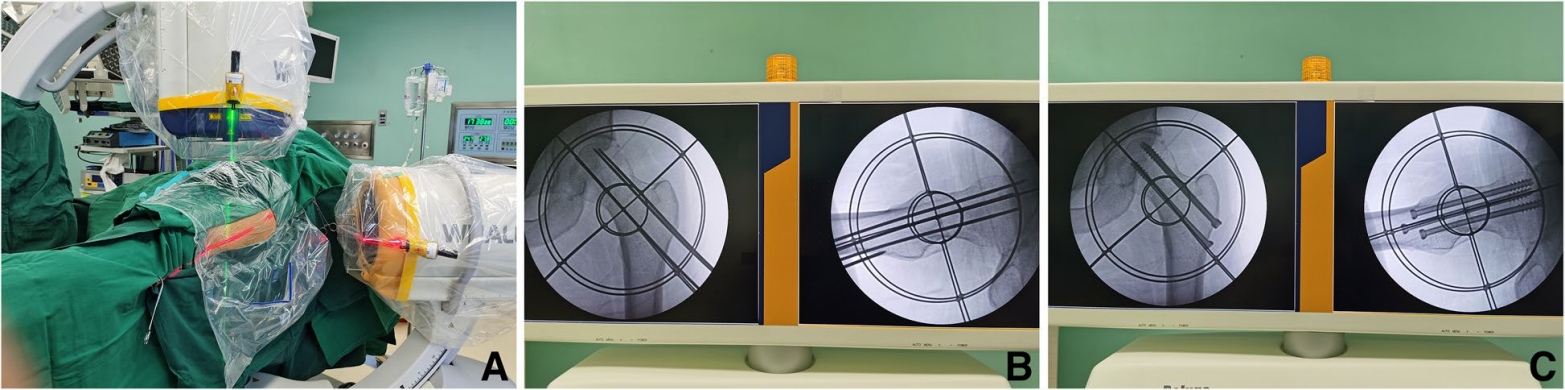
**A** The physical photos of the laser guiding navigation device. The horizontal red-light laser pointer and the coronal green-light laser pointer were fixed to the G-arm’s image intensifier by the special round strap. The angle of the laser pointer was determined by the position of the two positioning rings. **B** X-ray fluoroscopy confirmed that the Kirschner wires were inserted in the ideal position by laser. **C** X-ray fluoroscopy confirmed that the femoral neck cannulated screws were Inserted

All the patients were placed in the supine position on a special traction table using the G-arm machine. In the freehand group, the distal guide pin was first placed under the perspective of the G-arm machine; the other two guide pins were then placed with using the parallel drill guide. After accurate positioning, the screw channels were enlarged using a hollow drill along the guide pin, and three parallel cannulated screws (WASTON, Cannulated Compression Screws, diameter 7.3 mm) of suitable length were inserted for fixation. In the laser group, the positioning ring and laser emitter were calibrated before the operation; the patient was then anesthetized and disinfected, and the distal guide pin was inserted under laser-line guidance. The other two guide wires were also placed using the parallel drill guide. After the position was confirmed using fluoroscopy, three parallel cannulated screws were inserted in the same manner as in the freehand group (Fig. 4).

**Fig. 5:**
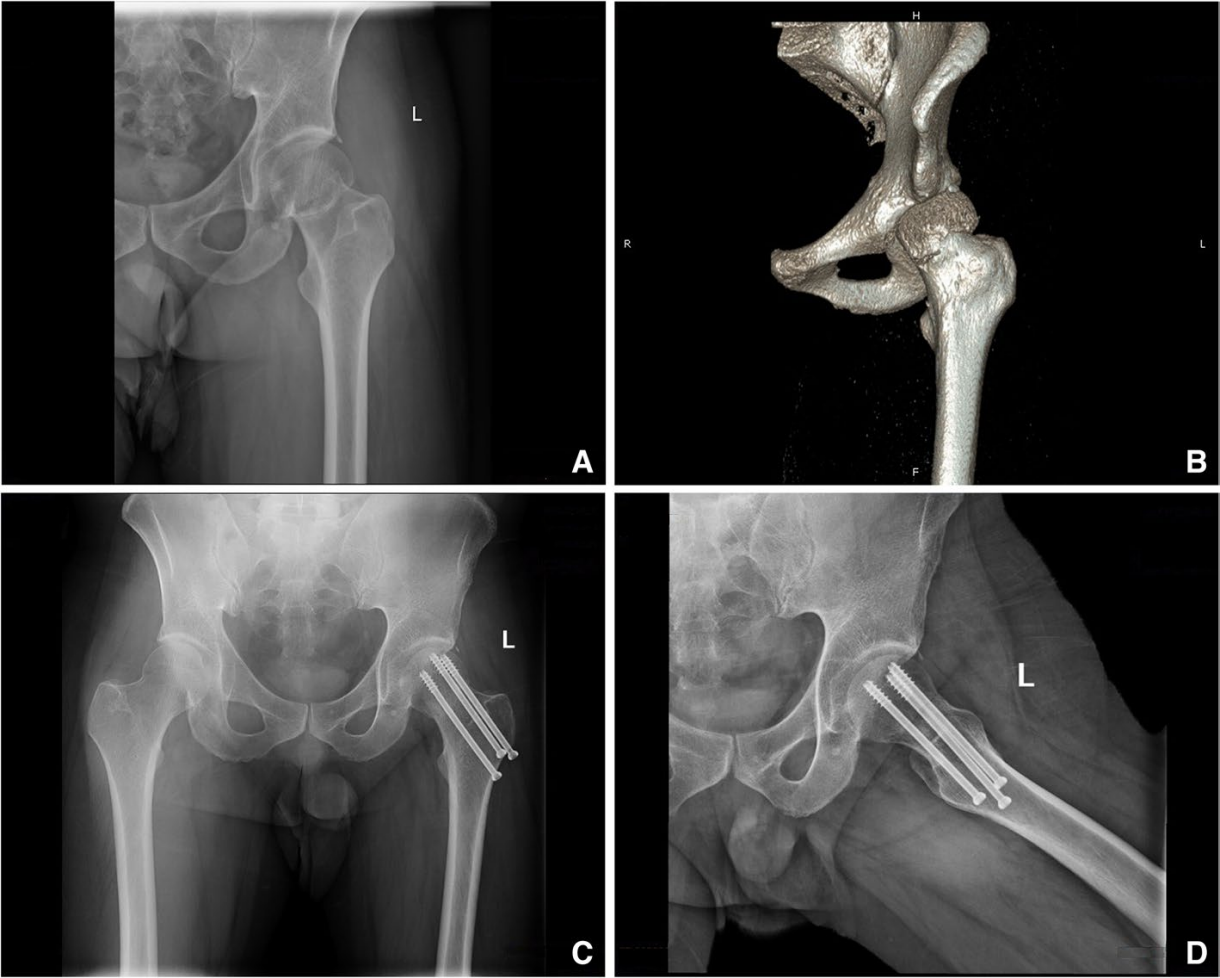
Preoperative X-ray and three-dimensional CT image of 39 years male with femoral neck fracture (**A**, **B**). X-ray image of this patient undertaken femoral neck cannulated screws with the laser guiding navigation device, and the fracture healed after operation 3 months later (**C**, **D**)

The operation time, radiation-exposure time, first success rate, amount of bleeding, visual analog scale (VAS) score, Harris score, and fracture-healing time were recorded and used for comparison among the two groups. All the patients were monitored at 1, 2, 3, 6, and 9 months postoperatively, or the follow-up ended after fractures healed (Fig. 5).

#### Statistical analysis

The study was designed to detect, with 90% power and an overall 5% Type I error rate, a 32 s decrement in operation time and 80 s decrement in radiation-exposure time from 172 s to 106 s, as estimated in the freehand group, based on the pilot in-vitro experiment. Thirty subjects were required for the final analysis.

The independent sample t-test, Wilcoxon rank-sum test, Chi-square test, and Fisher’s exact test were used to compare the outcomes between the two groups, where appropriate. All the statistical analyses were performed using the SAS JMP version 16.0 (SAS Institute) software, and statistical significance was defined as *P* < 0.05.

## Results

###  In-Vitro experiments

The average operative time of the freehand group was 177.5 s, whereas the time was 136.5 s in the laser group. The average radiation-exposure time of the freehand and the laser groups was 105.5 s and 27 s, respectively. All differences were statistically significant (Wilcoxon signed rank test, *P* < 0.05, Table 1).

**Table 1:** In-vitro Experiments data. Descriptive statistics for each group presented as Median (Lower Quartile; Upper Quartile), and Wilcoxon signed rank test was applied

Key data	Laser group	Freehand group	*Z*	*P* value
Operation time (Sec)	136.5 (133.8-142.3)	177.5 (151–198)	2.4245	0.0153
Radiation exposure time (Sec)	27 (21.25–32.25)	105.5 (102–109)	3.7475	0.0002

###  In-Vivo experiments

Among the 34 patients admitted to our hospital from June 1st, 2021 to April 1st, 2022, 32 patients who matched the inclusion and exclusion criteria were enrolled (Fig. 1). Sixteen patients were randomized to the laser and freehand groups each. Thirty patients completed the study period. Two patients were lost at postoperative follow-up (one patient in each group was excluded). Table 2 showed the characteristics of patients in the two groups. There was no statistically significant difference in age, sex, causes of injury, and fracture classification (*P* > 0.05).

**Table 2:** General characteristics of patients. Categorical data were compared using χ2 tests or Fisher’s exact test. There was no statistically significant difference in two group

Characteristics	Laser group (*n* = 15)	FH group (*n* = 15)	Fisher’s */χ2*	*P* value
Male	10 (66.7)	10 (66.7)	——	——
Female	5 (33.3)	5 (33.3)		
Age	57.73 (4.71)	58.6 (6.72)	0.41	0.68
**Cause of injuries**				
Traffic injuries	6 (40)	7 (46.67)	Fisher’s exact test	0.99
Fall injury	7 (46.67)	7 (46.67)		
Other	2 (13.33)	1 (6.67)		
**AO classification**				
B1	5 (33.33)	5 (33.33)	Fisher’s exact test	0.72
B2	8 (53.33)	6 (40)		
B3	2 (13.33)	4 (26.67)		
Follow-up(month)	12.33 (1.68)	11.93 (2.05)	-0.58	0.56

The first success rate was 93.3% (14/15) in the laser group and 60% (9/15) in the freehand group; the difference was statistically significant (χ2 = 4.658, *P* = 0.0309). The operation time of the laser group was 45 (40–45) min, whereas the time was 70 (65–75) min in the freehand group. The radiation-exposure time of the laser and freehand groups was 35 (35–40) s and 110 (95–125) s, respectively. The operation time of the laser group was significantly shorter than that of the freehand group (Z = 4.62991, *P* < 0.001), and the radiation-exposure time was significantly reduced in the laser group compared to that in the freehand group (Z = 4.68056, *P* < 0.001). The bleeding amount of the laser and freehand groups were 30 (20–35) mL and 50 (40–50) mL, respectively, and the difference was statistically significant (Z = 3.97943, *P* < 0.001). There was no statistical difference in VAS score, Harris score, or fracture-healing time between the two groups (Table 3).

**Table 3:** In-vivo Experiments Section data. Continuous variables with non-normal variables were reported as median (Lower Quartile; Upper Quartile)，and categorical data were analyzed using Fisher’s exact test or χ2 tests

Operative and postoperative data	Laser group (*n* = 15)	FH group (*n* = 15)	Z/*χ*^2^	*P* value
Operative time(min)	45 (40–45)	70 (65–75)	4.62	< 0.0001
Radiation exposure time(Sec)	35 (35–40)	110 (95–125)	4.68	< 0.0001
Amount of bleeding (ml)	30 (20–35)	50 (40–50)	3.97	< 0.0001
Preoperative VAS score	8 (8–9)	8 (8–9)	0.95	0.34
Postoperative VAS score	2 (2–2)	2 (2–2)	1.31	0.18
First success rate				
No	1 (6.67)	6 (40)	4.65	0.03
Yes	14 (93.33)	9 (60)		
Harris score	87 (85–89)	87 (85–89)	0.16	0.86
Fracture healing time(week)	13 (12–15)	14 (13–15)	0.89	0.37

## Discussion

This study evaluated the efficacy and safety of the laser-guiding navigation system in femoral neck-cannulated screw fixation. Based on Desargues’s projective geometry theorem, the target channel was visualized and located by the intercross laser line using special matching positioning ring. In the in-vitro experiments, we verified the advantages of the device based on the guide pins’ insertion and radiation-exposure time by comparison with the freehand group (Fig. 5).

**Fig. 6:**
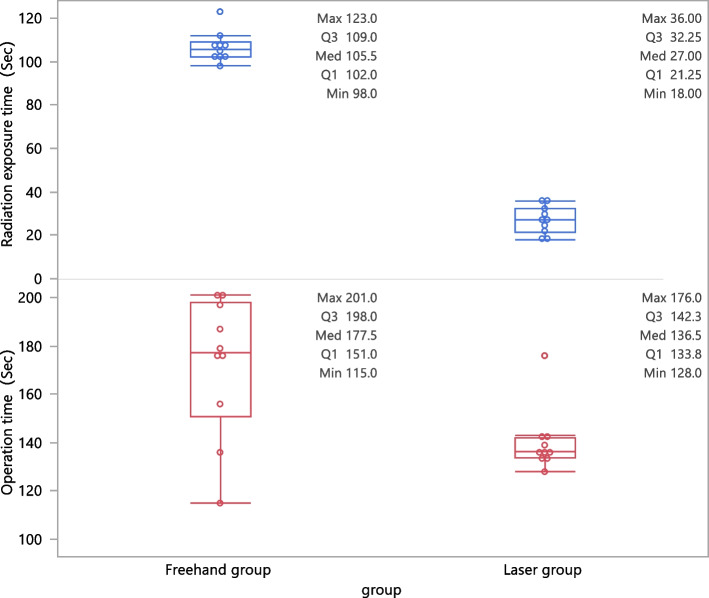
The box plot showed that the laser group was better than the FH group in terms of operation time and radiation exposure time, in-vitro experiments

In the in-vivo study, there was no statistical difference in VAS score, fracture-healing time, and Harris score between the laser and freehand groups. The laser group performed better in terms of operative time, radiation-exposure time, and operative blood loss, which can improve the efficiency of surgery (Fig. 6).

**Fig. 7:**
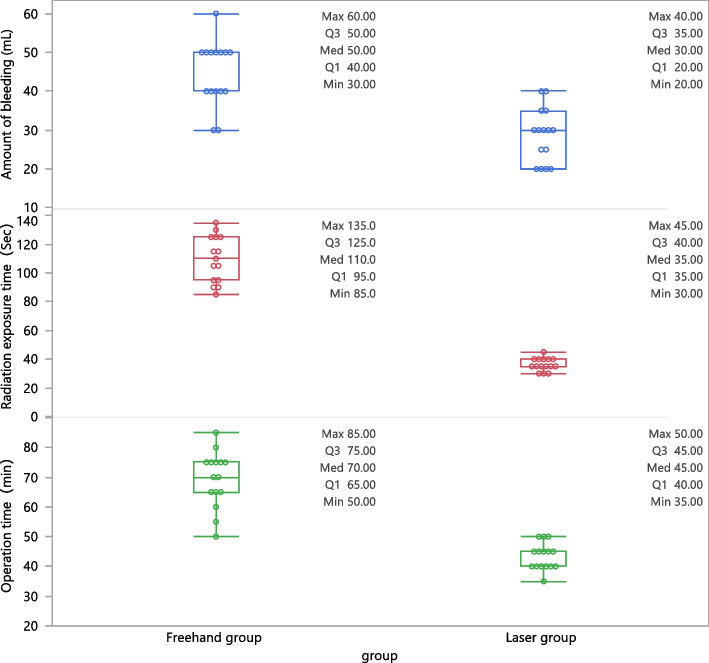
The box plot showed that the laser group was better than the FH group in terms of operation time and radiation exposure time and blood loss, in-vivo experiments

Through clinical the in-vivo study, the first success rate of the laser group could reach 93.3% vs. 60% in the freehand group, indicating a significant improvement. We further verified the characteristics of its high accuracy (Fig. 7), and provided a novel consideration for solving this technical problem.

Over the last decade, the field of orthopedic navigation has developed rapidly. New technologies have been invented to optimize the femoral neck-cannulated screw-inserted procedure. From the first generation, represented by instrument-positioning device [10–12], to the second generation, represented by optical navigation technology of orthopedic surgical robot [4, 13–20], they provide substantial help in orthopedic surgical navigation. However, they have the following drawbacks: cumbersome operation, expensive equipment, inaccuracy, and more radiation exposure (Table 4).

**Table 4:** Summary of published technologies of femoral neck cannulated screw insert procedure (2000–2021)

Study	years	Localization	Technology	Precision accuracy	First success rate	Operation time	Radiation exposure time
Browbank I [10]	2000	Femoral neck model	Mechanical devices based on X-ray	3.4 mm			
Schep NW [15]	2003	Femoral neck sawbones model	Fluoroscopy-based navigation Medivision system	1.17 mm			
D.Kendoff [16]	2006	cadaver femur specimen	Parallel drill guide (PDG)			25 ± 3 min	18 ± 2 s
Müller MC [11]	2011	cadaver femur specimen	computer-assisted navigation system based on 2D fluoroscopy		14/15	66.0 ± 22.2 min	4.5 ± 0.8 s
Wang JQ [13]	2011	Synbone hip models	Bi-planar robot navigation system (TINAV, GD2000)			26.39 min	8.35 s
Müller MC [3]	2012	Femoral sawbones model	Three-dimensional computer-assisted navigation			38.3 min	200s
Benjamin Moulin [17]	2019	FNF patients	ENS Imactis® CT navigation	8.0 ± 4.5 mm	96%	111 ± 51 min	
Duan SJ [4]	2019	FNF patients	Orthopaedic surgery robot TiRobot			62.6 ± 8.7 min	26.5 ± 7.4 times
Meng He [14]	2019	FNF patients	Bi-Planar Robot Navigation System	Error of 1.08° in the coronal plane and 1.25° in horizontal plane	99%	12.7 min	5.7s
Tong Yu [18]	2019	FNF patients	Three imensional computed tomography			57.3 min	6.3 times
Tomotoshi Murakami [19]	2021	Femoral intertrochanteric fracture patients	ADAPT system based on C-arm machine			28.3 ± 6.99 min	1.98 ± 1.40 min
Sizhe Wang [20]	2021	Femoral intertrochanteric fracture patients	3D-Printed Navigation Template	3.04 ± 0.39 mm	93.3%		

Among them, the key characteristics [21] of optical navigation are optical location tracking and image registration, which require technically demanding and dedicated equipment, more time registration, higher cost, and lower penetration [22]. Initially, accord to the Desargues’s theorem [23, 24], we wanted to design the laser-positioning navigation device, which could easily complete the intraoperative positioning accurately and is easy to promote, based on existing equipment [9]. After preoperative installation and debugging, the device can realize real-time calibration by the customized positioning ring, using G-arm. The accuracy and reliability of the device was verified in distal locking of femoral intramedullary nails [6]. Simultaneously, the freehand technique [25] is regarded as the gold standard, when the other methods do not work. Therefore, we selected the freehand technique as the control. Through in-vivo and in-vitro randomized controlled trials, it was proven that the novel device can improve the efficiency of surgery and reduce radiation time compared with freehand means, which provides a new idea for laser positioning and navigation.

Although the findings of this study are persuasive, there were limitations. First, the device could achieve precise location; however how to maintain the stability of guide needle in the process of drilling on non-vertical surface must be further improved. Second, the experience level of the two surgeons was comparable; it was impossible to exclude the bias caused by the difference in technical expertise. Third, although the number of cases had reached the sample size required by statistics, more cases in multiple centers would make the results more convincing.

## Conclusion

Compared with the freehand group, the laser-navigation group for the cannulated screw fixation of femoral neck has high surgical efficiency and less radiation-exposure time. This novel device facilitates the accuracy in completing the insertion of femoral neck-cannulated screws and provides a choice for improving the efficiency of surgery.

## Data Availability

This study was registered on Chictr.org.cn (23/05/2022, No. ChiCTR2200060236). All data are uploaded to the Clinical Trial Management Public Platform (medresman.org.cn). The datasets used and/or analyzed during the current study are available from the corresponding author on reasonable request.
